# A story-telling cloth approach to motivating cervical cancer screening in Mali

**DOI:** 10.3389/fpubh.2022.1051536

**Published:** 2022-12-15

**Authors:** Tiffani Crippin, Karamoko Tounkara, Eliza Squibb, Sarah Beseme, Katherine Barry, Kotou Sangare, Saidou Coulibaly, Pinda Fané, Aliou Bagayoko, Ousmane A. Koita, Ibrahima Teguété, Anne S. De Groot

**Affiliations:** ^1^GAIA Vaccine Foundation, Providence, RI, United States; ^2^GAIA Vaccine Foundation, Bamako, Mali; ^3^The Warren Alpert Medical School of Brown University, Providence, RI, United States; ^4^Laboratory of Applied Molecular Biology, University of Sciences, Techniques, and Technologies, Bamako, Mali; ^5^Reference Health Center of Commune 1, Bamako, Mali; ^6^Department of Regional Health, Bamako, Mali; ^7^Gabriel Touré Teaching Hospital, Bamako, Mali

**Keywords:** Human Papillomavirus, cervical cancer, West Africa, infectious disease, education, screening, vaccine, textile

## Abstract

Ninety percent of deaths from Cervical cancer (CC) caused by Human Papilloma Virus (HPV) occur in low- and middle-income countries. CC is the 2nd most common cause of cancer in women in West Africa, where 12,000 women develop cervical cancer and more than 6,000 die from the disease, annually. While HPV vaccination and CC screening have dramatically reduced the incidence of CC and mortality from CC in developed countries, prevention of CC in West Africa is often limited to visual inspection of the cervix and surgical intervention. In previous studies of CC in Mali, we demonstrated that knowledge about the link between HPV and CC is limited, and that screening for CC is often delayed until women are symptomatic. For this intervention, a story-telling cloth (West African-style printed pagne) was designed for use as a starting point for educational sessions run by community health workers. Community outreach using the cloth during 6 months of 2015 resulted in a 5-fold higher uptake of cervical cancer screening and increased awareness of the potential to vaccinate adolescents against CC. 3,271 women were motivated to visit one of five participating clinics for CC screening, where a mere 600 women had been screened during the previous year. This study shows that a comprehensive, visual, community-centered education campaign coupled with coordinated support for local clinics improves uptake of CC screening.

## Introduction

Cervical cancer (CC) is the fourth most common cancer in women worldwide. In 2018, 604,000 cases were diagnosed, and 342,000 women died from CC (1). Each year, 90% of these deaths occur in low- and middle-income countries (2). In Western Africa alone, 12,333 women developed CC in 2021, with 6,867 dying from the disease (3). The main risk factor of CC is infection by high-risk types of Human Papillomavirus (HPV). HPV types 16 and 18 cause 70% of cervical cancers and pre-cancerous cervical lesions. HPV vaccination and CC screening are routinely practiced in more developed countries and are an efficient method to reduce the incidence of CC. For example, in Western Europe where CC screening and national protocols to vaccinate the female population are available in all countries, the standardized incidence of CC is 10.7 per 100,000 woman age >15 years old (4). In Western Africa, however, the standardized rate of CC is double that, at 22.9 per 100,000 women (3).

In these low-income countries, knowledge about CC, HPV, and access to preventative measures are low or absent. Studies show that the risk of CC is directly correlated with education (5–7). One study shows that 21% of college-educated women have been screened for CC, 16% of women who completed secondary school receive screening, and only 8% of women with primary school education have been screened (2). Another barrier to successful HPV vaccination and CC screening campaigns is the lack of awareness about CC its relationship with HPV infection, which is driven by socioeconomic factors such as low income, low literacy, and low levels of education (8–11). As such, an intervention that does not presuppose literacy could be more effective in lower income urban and rural areas where literacy rates are lower (12).

Mali, a land-locked country in the heart of Western Africa is particularly hard hit by CC, with an age-standardized incidence as high as 36.4 cases per 100,000 woman aged > 15 years (3). While CC screening by visual inspection with acetic acid (VIA) is available in Mali, only an estimated 4.3% of eligible women received CC screening in 2018. HPV vaccination was introduced by GAVI (Global Alliance for Vaccines and Immunization) as a pilot program in 2016, and recent information suggests that national program could be in place as soon as 2023. HPV vaccination coverage of 90% is projected to decrease the risk of developing HPV-associated CC by 89% (13). In another study, vaccination of 50% of girls could reduce the peak prevalence of HPV 16/18 to only 5.0% in the urban setting and 9.6% in the rural setting, down from 11.7 and 22.0% respectively with no vaccination (14). This simple preventative measure, combined with higher screening rates could drastically decrease the incidence of CC in Mali and similar countries.

In 2011, working in close partnership with Malian scientists, doctors, and public health officials, GAIA Vaccine Foundation (GAIA VF), a US-based nonprofit organization, initiated a study to examine knowledge, attitudes and practices (KAP) related to HPV and CC with a survey in two neighborhoods in Bamako (15). In parallel, GAIA VF worked with Malian clinicians and researchers to determine that 83% of the HPV strains found in CC biopsies are vaccine-preventable high-risk HPV strains (16). A key finding from these studies was that knowledge of HPV and its link to CC was extremely low; <8% of the 301 male and female participants knew about the connection between HPV and CC. In addition, CC screening was nearly non-existent with only 5% of female study participants having previously received screening. Similar data have been found by independent research (17).

The cost of CC screening ($2–3/test) and low literacy are two major barriers to increasing CC screening rates in Mali. In 2018, 77% of Malian adult females were illiterate (18). The lack of trained health care personnel and shortage in acetic acid (AA) and Lugol iodine (LI) (which are used in CC screening with the method of visualization with a colposcope [VIA, VILI]) were also identified as barriers to providing routine CC screening in CHCs (19).

Building on previous results and to address these barriers, a comprehensive community-led education campaign using a West African-style textile pattern—the “Story-telling Cloth”—to inform communities about prevention methods was launched in five community health centers (CHC) in Bamako in 2015. The surveys carried out during the campaign measured the impact of the intervention on the number of women accessing CC screening, their knowledge, awareness, and practices regarding the underlying causes and the preventative measures of CC, as well as their willingness and preferences to vaccinate their daughters.

## Materials and methods

### Collaborative preparation of the HPV/CC awareness campaign and design of the story-telling cloth

A comprehensive community-led education campaign using a West African-style textile pattern—the Story-telling Cloth—was developed, with the goal of informing women in Commune 1 of Bamako about the cervical cancer and the availability of cervical cancer screening at five community health centers (CHC) in Bamako in 2015 with funding from the Gates Foundation. As part of this program, support was provided to train health care personnel in CC screening protocols and to offer CC screening for free at the participating CHCs. Educational radio announcements were broadcast concurrently to promote CHC services. Surveys were performed to measure the impact of the outreach intervention. We assessed whether the campaign clarified and emphasized the link between CC and HPV infection, and whether the story-telling cloth approach was an effective method for informing the community about the availability of cervical cancer screening at local clinics.

The protocol design, surveys, and consent forms were reviewed and approved by a US-based institutional review board (Ethical and Independent Review Services) on March 23, 2015 (E and I Project#15010) and by the local ethics review board of the UTTSB (University of Bamako). The objectives, benefits, and risks of participating in the study were explained to the patients at the CHCs in Bambara or French. Informed consent was obtained, as per approved protocol. The six-page survey is provided in Supplementary material S1.

GAIA VF worked with experienced clinicians and public health experts in Mali to develop and implement the intervention. First, discussions were held with public health officials to determine infectious disease prevention priorities in Mali. Then, GAIA VF sought feedback and active participation from public health partners for the development of the program. The Regional Department of Health (DRS) identified community health centers (CHCs, the first level of health structures in Mali) with qualified staff serving a large population that will benefit the most from the program. Discussions were initiated with the physician director of each CHC, who identified staff to participate in the program.

**Figure 1:**
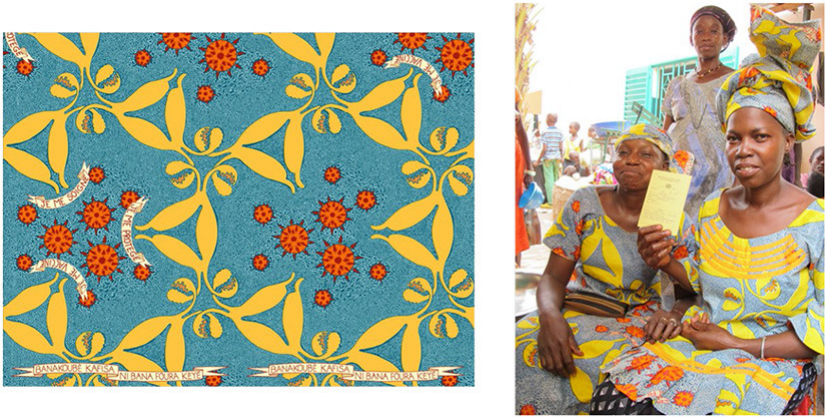
Story-telling cloth for cervical cancer prevention. The photo on the **left** shows the story-telling cloth pattern. It illustrates the HPV virus, cervix, uterus, and fallopian tubes. Healthy cells surround each cervix and transform into cancerous cells as they encounter the HPV virus. The blue background depicts healthy cells surrounding each cervix that transform into cancerous cells as they encounter the HPV virus. The photo on the **right** shows two women wearing an outfit made with the fabric showing their CC screening card, obtained after a first CC screening as a reminder to come back yearly (Permission given for images).

With feedback from these collaborators, the story-telling cloth was designed by members of the study team (ADG and ES). It drew inspiration from West African commemorative prints that are often used to promote events such as International Women's Day and World AIDS day. It was also designed to act as a teaching tool used by community health workers during outreach events. The pattern illustrates the HPV virus, cervix, uterus, and fallopian tubes. The blue background depicts healthy cells surrounding each cervix, these transform into cancerous cells as they encounter the HPV virus. The pattern contains a slogan in French, “I protect myself, I care for myself, and I get vaccinated” and a local proverb in Bambara, Banakoubé kafisa ni bana foura kèyé, meaning, “It is better to prevent than to cure” (Figure 1). Printed in a local factory, the fabric was distributed in the community and was used to sew outfits, handbags, hair bands or other wearable items. Figure 1 shows the pattern, and two women wearing an outfit made with the fabric, holding their CC screening cards.

Training was completed between March 30 and April 4, 2015. The outreach session and cervical cancer screening ran for 6 months from April 15 to October 15, 2015. Education sessions were held twice a week in the communities over those 6 months and radio shows promoted the outreach weekly. Surveys were given to the first 100 women seeking treatment at each site.

### Location of the intervention

The campaign was designed to reach women and families across four neighborhoods of Commune 1 in Bamako, within four CHCs and one referral health center (RHC, which features more qualified staff and technical resources than CHCs). At each of these five sites, healthcare practitioners promoted CC screening while wearing the story-telling cloth. Education sessions were led within the clinics by midwives and nurses, as well as in the neighborhoods surrounding the clinics by community health workers (CHW). The outreach sessions were associated with neighborhood gatherings of women called “grains” that take place on a weekly basis in Commune 1. Both the healthcare personnel and the CHW who provided these education sessions were all adult women, of the same age as the target population. The outreach workers initially attended a 3-day training session led by representatives of the Regional Health Department and the RHC.

A participative methodology was used, and the topics covered included the female reproductive system, precancerous lesions of the cervix and key messages relative to CC prevention. Methods and tools of communication adapted to this health topic in the local context were also covered, as well as the means to use the story-telling cloth to dispense information within the members of the community. The report summarizing the training is available in French in Supplementary material S2.

The story-telling cloth was the focal point of the outreach education sessions, which included information about HPV, CC, and CC screening and where to access these health services. Women leading the sessions wore outfits made from the story-telling cloth and objects like handbags or the fabric itself were distributed during the sessions. The launch of the campaign was televised, and announcements and discussions were broadcasted on radio and TV stations.

### Cervical cancer screening and follow-up

Visual inspection with acetic acid (VIA) and Lugol iodine (VILI) are protocols in place for CC screening in CHCs and referral centers. VILI is a confirmation test that is performed at the referral site (RHC) in the event of a positive test with VIAA. The program supported by GAIA VF included capacity building in the CHCs by funding training of nurses and midwives to the VIA screening methods and provided a steady supply of acetic acid and Lugol iodine, chemicals used in VIA and VILI.

A total of 15 nurses and midwives at all five sites were re-trained on CC screening protocols by Regional Department of Health agents. CC screening was offered at no cost at the five clinics.

Women with a normal CC screening were provided with a screening card and a scheduled date for an appointment the next year per Malian protocol. Women with abnormal results on the first screening were referred to the RHC to receive a confirmation test. In case of a double positive screening, a biopsy was taken during a follow up visit at the RHC, and final diagnosis was established within 2 weeks. Confirmed cases of precancerous and cancerous lesions were referred to the Obstetrics and Gynecology department of the central city hospital Gabriel Touré for observation, cryotherapy or LEEP (Loop Electrosurgical Excision Procedure).

### Data collection and statistics

A survey questionnaire was proposed to the first 100 women seeking screening at each health center (total number of women: 500, all aged 18 years old or more). The survey, adapted from GAIA VF's previous studies, was administered verbally by a trained nurse, and was designed to determine the effectiveness of each communication method on motivating women to receive CC screening and assess knowledge, attitudes and practices (KAP) toward HPV and CC. As HPV vaccination was not available at the time of this project, the survey also contained questions to estimate the participants' willingness to include their daughters in an HPV vaccination program. Survey data was coded into Microsoft Excel by a trained data input technician by hand. All data was analyzed using Stata version 16.1.

### Statistical analysis

Table 1 shows the descriptive statistics for one of the main dependent variables, *knowledge*, and each of its component variables. To create the variable *knowledge*, each participant received 1 point per component question allowing for a minimum of 0 and a maximum of 7 points. For example, if the participant answered “Yes” to “Do you know what an STI is?”, then they received 1 point for *knowledge*. If they answered “No”, then they would receive 0 points for that component. A participant who answered “Yes” to all of the component questions would receive a 7 on the *knowledge* variable. The results of our statistical analysis remain substantively unchanged when this variable is created through factor analysis^1^, suggesting that our simple operationalization is robust to alternative specifications.

**Table 1:** Descriptive statistics for knowledge variable.

**Knowledge variable**	***N***	**Mean**	**St. Dev**	**Min**	**Max**
Knowledge	500	3.17	1.77	0	7
**Components**					
Do you know what an STI is?	500	59%	0.492	0	1
Do you know what HPV is?	500	13%	0.332	0	1
Have you heard of CC?	500	75%	0.436	0	1
Do you know the causes of CC?	500	28%	0.449	0	1
Do you know the symptoms of CC?	500	22%	0.417	0	1
Do you agree that CC causes death?	500	75%	0.433	0	1
Do you agree that HPV causes CC?	500	45%	0.498	0	1

Ranges for all variables included in the Min & Max Columns.

STI, Sexually Transmitted Infection; HPV, Human Papillomavirus; CC, Cervical Cancer.

To evaluate the impact of the outreach sessions on women's knowledge about HPV and CC we use linear regression analysis with robust standard errors clustered by health center. Although the dependent variable is ordinal which suggests the use of an ordered logistic regression model, the model fails to converge on the Brant Test of the Parallel Regression Assumption due to small sample size and limited variation in the data. For this reason, we present the linear regression output in the main text while the results of the ordered logistic regression models can be found in the Supplementary material S3. Fortunately, our substantive results remain consistent across both model specifications. We also present results both with and without fixed effects by health center to control for possible unmeasured variation between health centers.

We use maximum likelihood estimation probit models to predict (1) the likelihood of wanting the HPV vaccine in Mali and (2) the likelihood of the participant wanting their own child to be vaccinated since the dependent variables are binary. Linear regression assumes a continuous dependent variable with normally distributed errors and linear relationship between the covariates, which is not the case with binary dependent variables. Using linear regression on binary dependent variables leads to nonsensical results like probabilities >1. For this reason, we use probit regression, which uses a nonlinear function to model the conditional probability of each outcome in the dependent variable. Finally, both models used fixed effects by health center and robust standard errors to account for unmeasured heterogeneity.

## Results

Table 2 shows the descriptive statistics for all variables used in later models. The mean age of participants was 31.3 years old, and while 53% of the participants were among the WHO target age range for CC screening (30–49 years old) (20), most of them were within the age range defined in guidelines for CC screening in Mali (20–65 years). The age at first sexual intercourse was consistent with the latest data available (21). Most participants were married and 38% were in a polygamous marriage. Of note, as much as 43% of participants had not received any schooling, suggesting that visual education tools, as opposed to written ones, should be strongly considered when developing educational materials targeting women in Mali. Most of the participants had children (91%) and many of them had received a vaginal exam (76%). While the number of women who had previously been screened for CC was low (16%), this cohort still had more CC screening than the national average, which is about 3% (22).

**Table 2:** Descriptive statistics for women participating in the study.

**Demographics**	***N***	**Mean**	**St. Dev**	**Min**	**Max**
Age	487	31	9.83	15	70
Married, not polygamous^a^	500	54%	0.5	0	1
In polygamous marriage^a^	500	38%	0.49	0	1
Single, cohabitating, divorced, widowed^a^	500	9%	0.28	0	1
Any Schooling	500	57%	0.5	0	1
Level of Schooling^b^	500	2	1.78	0	5
**Medical history**					
Ever been pregnant	499	91%	0.29	0	1
Ever have vaginal exam	500	76%	0.429	0	1
Ever tested for HIV	500	77%	0.419	0	1
Had an STI	500	54%	0.5	0	1
Ever been screened for CC	500	16%	0.363	0	1
Know someone who has/had CC	500	15%	0.359	0	1
**Outreach**					
Attended an education session	500	32%	0.467	0	1
Saw new story-telling cloth	500	36%	0.479	0	1

Ranges for all variables included in the Min & Max Columns.

STI, Sexually Transmitted Infection; CC, Cervical Cancer.

^a^Categorical variable: ^b^Defined as 0 = no schooling, 1 = preprimary schooling, 2 = primary schooling, 3 = secondary schooling, 4 = tertiary schooling, 5 = more than tertiary schooling.

### Preference for the story-telling cloth campaign

The two major components of the story-telling cloth campaign were the cloth, consisting of a wearable imagery illustrating the link between HPV and CC, and the education sessions, which were led by community health workers (CHW) within the community health centers (CHCs) or at community gathering locations.

Education sessions are a popular way to dispense information about health. Most participants in this study (93%, *N* = 463) stated that they would like more education sessions for health issues such as child health (57%, *N* = 284), reproductive health (46%, *N* = 230), viral infections (46%, *N* = 229) or family planning (38%, *N* = 191) (Table 3).

**Table 3:** Participants' desire for education sessions, *n* (%).

**Would you like more education sessions for health issues?**	**463 (93%)**
Child health	284 (57%)
Reproductive health	230 (46%)
Viral infections	229 (46%)
Family planning	191 (38%)

Each row is a topic given by the survey participant as a health issue she would like to see more education sessions for.

### The story-telling cloth campaign and knowledge about HPV and CC

The primary goal of the story-telling cloth campaign was to raise awareness and increase knowledge about CC and the means to prevent the disease. Table 4 displays our main empirical findings. Model 1 shows our results without fixed effects and Model 2 includes fixed effects by health center. The results of both models support our hypothesis that attendance at an education session and viewing of the story-telling cloth increased women's knowledge of HPV and CC. The positive coefficients mean that the independent variable has a positive effect on knowledge and negative coefficients mean they have a negative effect. Since this is a linear regression model, the coefficients themselves can also be interpreted directly. For example, 1-point change in *Attended an Education Sessions* results in a 0.696 increase in *knowledge*. Similarly, a 1-point change in *Level of Schooling* results in a 0.211-point increase in *knowledge*, so the difference between no education (a 0 on *Level of Schooling*) and having more than a tertiary education (a 5 on *Level of Schooling*) results in a 1-point increase in *knowledge* (0.211 x 5). Unsurprisingly, level of schooling and many of the medical history variables are also statistically significant predictors of more knowledge about HPV and CC.

**Table 4:** Predicting knowledge about CC and HPV.

	**Model 1**	**Model 2**
	**DV = Knowledge**	**DV = Knowledge**
Attended an education session	0.696*	0.703*
	(0.305)	(0.311)
Saw story-telling cloth	0.748**	0.689*
	(0.241)	(0.259)
Age	0.005	0.009
	(0.009)	(0.009)
Married—monogamous^a^	−0.288	−0.296
	(0.316)	(0.312)
Married—polygamous^a^	−0.213	−0.232
	(0.430)	(0.417)
Level of schooling	0.211*	0.218*
	(0.078)	(0.081)
Ever been pregnant	−0.0619	−0.0981
	(0.232)	(0.204)
Ever had STI	0.574*	0.585*
	(0.247)	(0.262)
Ever been tested for HIV	0.565*	0.551*
	(0.220)	(0.225)
Ever had a pelvic exam	−0.240	−0.294
	(0.303)	(0.339)
Ever been screened for CC	0.915**	0.869**
	(0.231)	(0.248)
Know someone who had/has CC	0.805***	0.817***
	(0.137)	(0.146)
ASACOBOUL1^b^		0.077
		(0.151)
ASACODJE^b^		0.343**
		(0.0824)
ASACOMSI^b^		−0.015
		(0.149)
CSREF (RHC)^b^		−0.096
		(0.122)
Constant	1.601**	1.514**
	(0.451)	(0.408)
Observations	487	487
R-squared^+^	0.316	0.322

^+^ In this table, the R-squared value describes the amount of variation in the dependent variable that is explained by the model (or combination of independent variables). Model 1 explains 31.6% of the variation in the dependent variable and Model 2 explains 32.2%.

Method = Linear Regression. DV coded 0 to 7 from less to more knowledge.

ASACCOBOUL1, ASCODJE, ASACOMSI, CSREF (RHC), and ASACOBA are all the names of the participating health centers. Their inclusion as variables is to control for unobserved variance between centers/communities.

^a^The excluded group for these variables is “Unmarried”. ^b^The excluded group for these variables is ASACOBA.

Robust standard errors clustered by site in parentheses.

^***^p < 0.01, ^**^p < 0.05, ^*^p < 0.1.

Interpretation: The positive coefficients mean that the independent variable has a positive effect on knowledge and negative coefficients mean they have a negative effect. Since this is a linear regression model, you calculate the effect on the dependent variable by multiplying a change in the independent variable by the coefficient. For example, a 1-point change in Ever been Screened for CC results in a 0.915 increase in knowledge.

**Figure 2:**
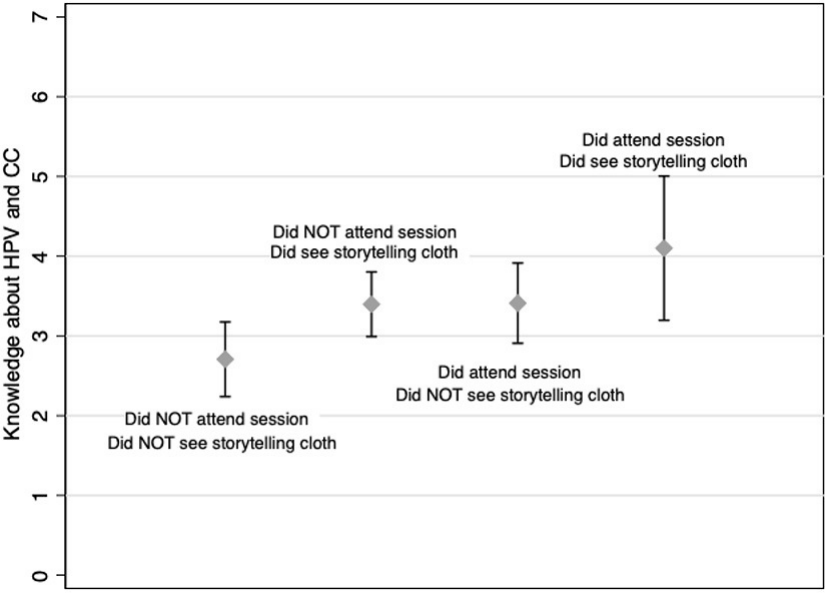
Predicted knowledge level by education session attendance and story-telling cloth viewing. Knowledge about STIs, cervical cancer and HPV. Study participants (*N* = 500) were asked if they knew what an STI (sexually transmitted disease) was and questions assessing their knowledge about CC and HPV were asked. The relationship (using the regression described in the text) for each of the variables under study is reported in the graphs for all participants. 95% confidence intervals are illustrated for each category. This graph demonstrates women who had neither attended an education session nor seen the story-telling cloth only scored a 2.7 on the knowledge scale, while women who had both attended a session and seen the story-telling cloth scored 4.1 on average. Importantly, there is no overlap in the confidence intervals of these two predictions suggesting that overall knowledge of CC and the link between HPV and CC was increased by exposure to the story-telling cloth campaign.

Figure 2 shows the impact of our two main variables (attendance at an education session and whether or not the survey participant had seen the story-telling cloth) on a woman's knowledge level. Holding all other variables constant, women who had neither attended an education session nor seen the story-telling cloth only scored a 2.7 on the knowledge scale, while women who had both attended a session and seen the story-telling cloth scored 4.1 on average. Importantly, there is no overlap in the confidence intervals of these two predictions leading us to conclude that overall knowledge of CC and the link between HPV and CC was increased by exposure to the story-telling cloth campaign.

### CC screening during the story-telling cloth campaign

**Figure 3:**
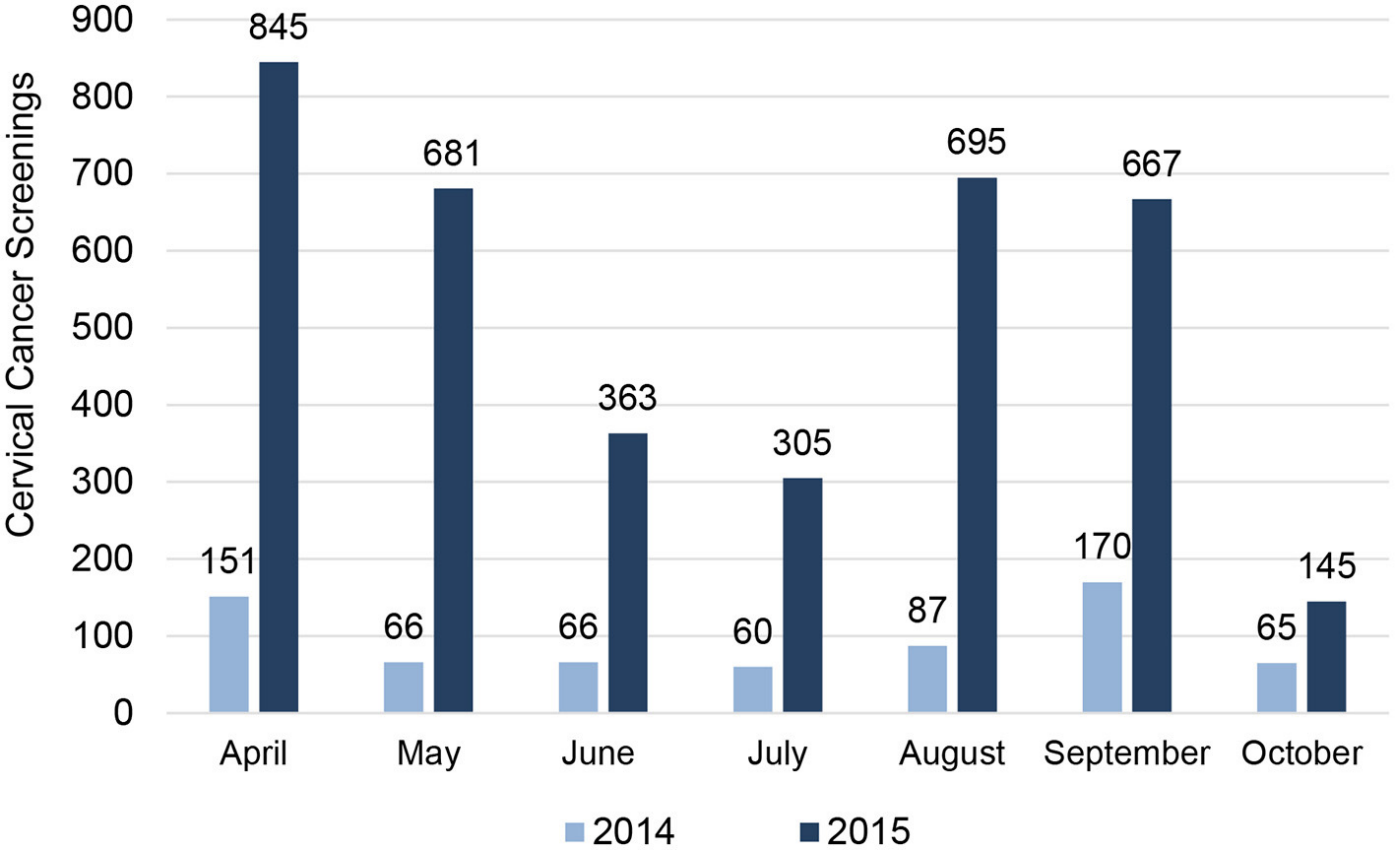
Comparison of CC screenings at partner sites by Month in 2014 and 2015. There was a five-fold increase in CC screens during the intervention. 3,701 women were screened from April through October 2015 (bars in dark blue), while over the same period in 2014, only 665 screenings were provided at all five sites (light blue bars). The histogram provides a month-by-month report for CC screenings at partner sites. Notable decreases in screens are seen during the months of Ramadan.

During the duration of this program, the uptake of CC screening increased almost 5-fold across the five participating clinics, when compared to data obtained during the same months of the previous years. Data from 2014 were obtained from the Regional Department of Health. Figure 3 shows that 3,701 women were screened from April through October 2015, while over the same period in 2014, only 665 screenings were provided at all five sites. Of the 3,701 women screened, 5% (*N* = 168) had a positive result after VIA. Unfortunately, only 58% (*N* = 97) of the women who tested positive with VIA visited the RHC for a confirmation test with VIL. The confirmation test was positive for 46% (*N* = 45) of women, 69% (*N* = 31) agreed to a biopsy, and all were treated according to best practices, typically with either colposcopy, cryotherapy, or LEEP resection.

**Figure 4:**
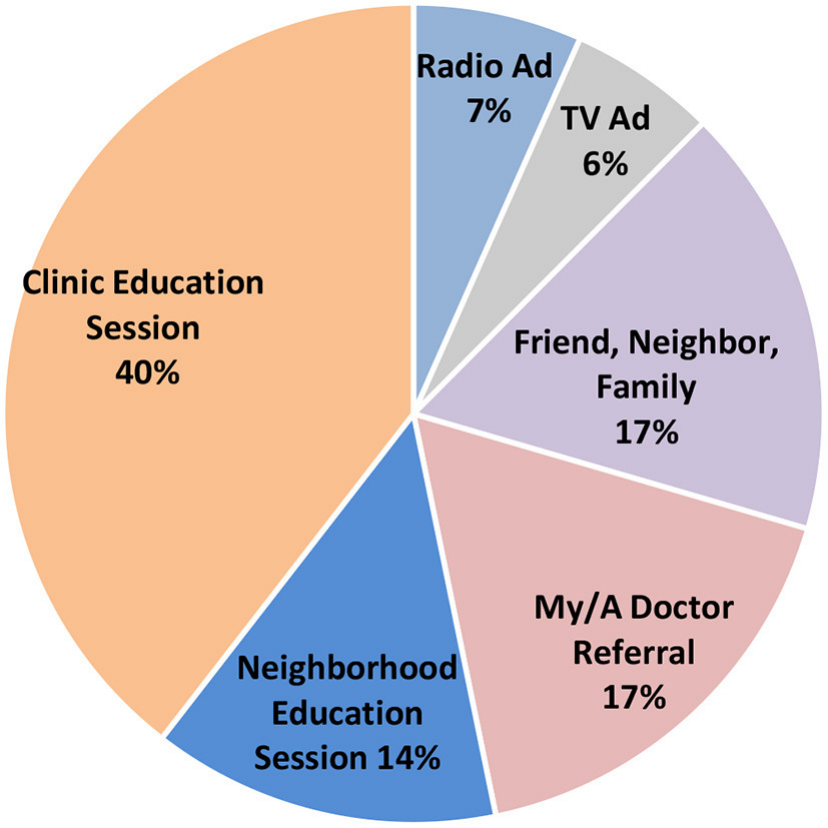
Reported motivation to get CC screening. This pie chart shows the distribution of reasons women gave for coming to the clinic to get screened for cervical cancer. Story-telling cloth sessions were held at the clinic sites and in the neighborhoods. In addition to “referral from a my/a doctor”, the educational sessions were the primary source of referrals for CC screening.

It is anticipated that increased knowledge about a disease increases demand for health services related to this disease. When asked what influenced them to come in for a CC screening, 53% (*N* = 258) of women answered the educations sessions, either in the clinic or in their neighborhood (Figure 4). TV and radio ads, two components of the campaign broadcast only at the beginning of the study were the reason for 87 women (13% of the total participants) to come in for a screening. Importantly, the survey did not allow for a distinction between ads from the story-telling cloth campaign or public announcements from the government-sponsored campaign. Recommendation from physician or word of mouth was the reason for 34% (*N* = 171) of participants. Note that the total number equals 516 because some women answered that multiple options motivated them.

### Efficacy of education session in delivering health message

While the same number of sessions were held in the CHCs and in neighborhood locations, education sessions at the clinic were the most effective at encouraging women to get screened: among women who attended education sessions (*N* = 258), 74% (*N* = 190) said that the session took place in their CHC and 26% (*N* = 68) in the neighborhood (Figure 4).

Table 5 presents a series of *t*-tests evaluating the difference in means between survey participants who attended an education session themselves, either in their CHC or neighborhood, (*N* = 160) and those who did not (*N* = 340). Each row gives the mean value for participants who attended a session, the mean value for participants who did not attend a session, the difference in the two mean values, then the *p*-value for the statistical significance of that difference for a difference variable of interest. Every difference in means is statistically significant at the 99% confidence interval (*p* < 0.01). Attendance statistically improved not only a participant's probability of seeing the story-telling cloth pattern (difference in means = 0.685; *p* < 0.01), but also their evaluation of it as an educational method (difference in means = 0.626; *p* < 0.01) and their likelihood of sharing the information in the pattern with their family, friends, and neighbors (difference in means = 0.623; *p* < 0.01). Survey participants who attended a session were also more likely to find the information about HPV and CC from the education sessions interesting (difference in means = 0.739; *p* < 0.01), share that information with their network (difference in means = 0.642; *p* < 0.01), and, perhaps most importantly, recommend their family, friends, and neighbors receive a CC screening (difference in means = 0.598; *p* < 0.01).

**Table 5:** Difference in means tests.

	**Attended a session**	**Did not attend a session**	**Difference in means**	***P*-Value**
Came in for Screening	*N =* 160	*N =* 340		
Have you seen the story-telling cloth pattern?	0.838 (0.029)	0.153 (0.020)	0.685 (0.035)	*p <* 0.01
Thinks the story-telling cloth method is effective	0.95 (0.017)	0.324 (0.025)	0.626 (0.039)	*p <* 0.01
Would or did share story-telling cloth info	0.944 (0.018)	0.321 (0.025)	0.623 (0.039)	*p <* 0.01
Thinks info from session is interesting	0.963 (0.015)	0.224 (0.023)	0.739 (0.035)	*p <* 0.01
Willing to share information about HPV & CC	0.963 (0.015)	0.321 (0.025)	0.642 (0.038)	*p <* 0.01
Would recommend CC screening	0.969 (0.014)	0.371 (0.026)	0.598 (0.039)	*p <* 0.01

All variables are coded 0/1 where 0 = No and 1 = Yes.

Standard errors in parentheses under means.

Attendance at an education session had no statistically significant impact on a participant's ability to interpret the cloth correctly. During the survey, participants were given a list of prompts to describe the information depicted on the pattern. Eighty percent of them correctly chose “Virus attacking the cervix”, “Cancer attacking the uterus”, or “Female organs and infection.” There was no statistically significant difference in the answers between participants who had attended a session and those who had not. This is ideal as it suggests that either (1) women spoke to each other about the cloth outside of the sessions or (2) the story-telling cloth is independently interpretable; both of which were goals for the intervention. Indeed, 94% of women who attended a session answered that they would or did share the information they learned from the story-telling cloth with their family, friends, and neighbors.

### HPV vaccine acceptance and vaccinations preferences

This survey also aimed to capture women's desire to vaccinate their daughters with the HPV vaccine. Table 6 shows the likelihood of wanting the HPV vaccine in Mali (Model 1) and the likelihood of the participant wanting their own child to be vaccinated (Model 2). Importantly, the data is biased positively toward the HPV vaccine with 93% (*N* = 463) wanting it available in Mali and 87% (*N* = 437) wanting their child to be vaccinated. Yet, more knowledge about HPV and CC still significantly improves the acceptability of the vaccine in general (Model 1) and specifically for the participant's children (Model 2). Positive coefficients mean that the independent variable increases the predicted probability of a 1 in the dependent variable (the probability that they survey participant answered yes to the question) and negative coefficients mean the variable decreases the predicted probability of a 1.

**Table 6:** Probit models evaluating the impact of knowledge on vaccine desirability.

	**Model 1**	**Model 2**
	**DV = Want HPV vaccine to be available in Mali**	**DV = Want my child to receive HPV vaccine**
Knowledge	0.213***	0.0829*
	(0.0521)	(0.0461)
Age	−0.0111	0.0150
	(0.0101)	(0.0101)
Level of schooling	−0.0343	−0.00701
	(0.0488)	(0.0487)
Married—Monogamous^a^	0.114	−0.233
	(0.360)	(0.307)
Married—Polygamous^a^	0.108	−0.370
	(0.365)	(0.317)
Have children	−0.234	0.440
	(0.405)	(0.283)
ASACOBOUL1^b^	0.0836	0.755***
	(0.257)	(0.235)
ASACODJE^b^	−0.297	−0.0159
	(0.246)	(0.214)
ASACOMSI^b^	0.538*	1.057***
	(0.326)	(0.314)
CSREF (RHC)^b^	0.244	0.257
	(0.254)	(0.235)
Constant	1.294***	0.00784
	(0.478)	(0.416)
Observations	486	486
Log pseudolikelihood	−118.93	−166.04

Method: Maximum Likelihood Estimation. DVs are coded as 0 = No 1 = Yes.

ASACCOBOUL1, ASCODJE, ASACOMSI, CSREF (RHC), and ASACOBA are all the names of the participating health centers. Their inclusion as variables is to control for unobserved variance between centers/communities.

^a^The excluded group for these variables is “Unmarried”. ^b^The excluded group for these variables is ASACOBA.

Robust standard errors clustered by site in parentheses.

^***^p < 0.01, ^*^p < 0.1.

**Figure 5:**
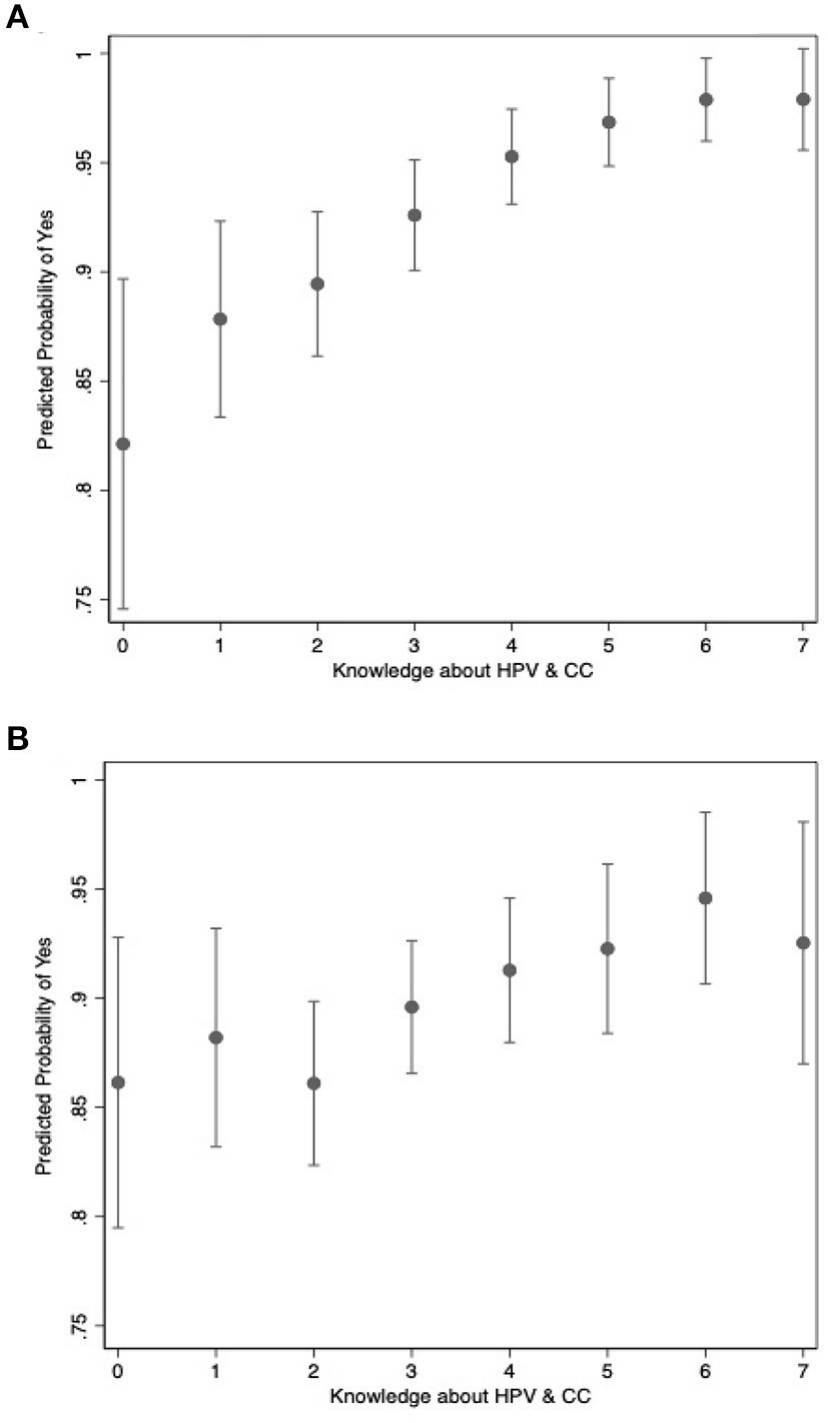
Relationship between knowledge and motivation. **(A)** Wanting the HPV vaccine to be available in Mali. Shows the predicted probability that a participant answers “yes” to whether they would like the HPV vaccine to be available in Mali, based on the level of knowledge calculated using the maximum likelihood model described in the text. **(B)** Wanting to vaccinate your own daughter. Show the predicted probability that a participant answers “yes” to wanting their own child to participate in an HPV vaccination campaign, based on the level of knowledge calculated using the maximum likelihood model described in the text.

Interpretation: Positive coefficients mean that the independent variable increases the probability of a 1 (or a yes in this case) in the dependent variable and negative coefficients mean they decrease the probability of a 1. The coefficients themselves are not independently interpretable without transformation like in Figures 5A,B.

The coefficients themselves cannot be independently interpreted like in linear regression, but their effects are displayed in the marginal effects' graphs below. Figures 5A,B show the predicted probability that a participant answers “yes” to whether they would like the HPV vaccine to be available in Mali (Figure 5A) and like their own child to participate in an HPV vaccination campaign (Figure 5B). These probabilities are plotted over the range of knowledge about HPV and& CC. The predictions themselves are plotted as dots and 95% confidence intervals are depicted by the capped vertical lines. Knowledge about HPV and CC has a large and positive effect on the probability that a participant wants the HPV vaccine to be available in Mali increasing the probability of the participant answering yes from about 83% to nearly 100%.

Of the women who said that they would vaccinate their daughter, 368 gave a specific reason for desiring vaccination: Among them, 76% (*N* = 278) mentioned “Prevention of CC”, 13% (*N* = 47) mentioned the exact proverb depicted on the story-telling cloth “It's better to prevent than cure”, and 12% (*N* = 43) that responded mentioned the word “Protection”.

Interestingly, only 13% (*N* = 60/463) of participants who wanted the vaccine available in Mali and 13% (*N* = 58/437) of participants who wanted their child to be a part of a vaccination campaign also answered “yes” to “Do you know what HPV is?” suggesting that the positive bias is the result of a positive opinion on vaccination in general rather than a specific response to our campaign.

**Figure 6:**
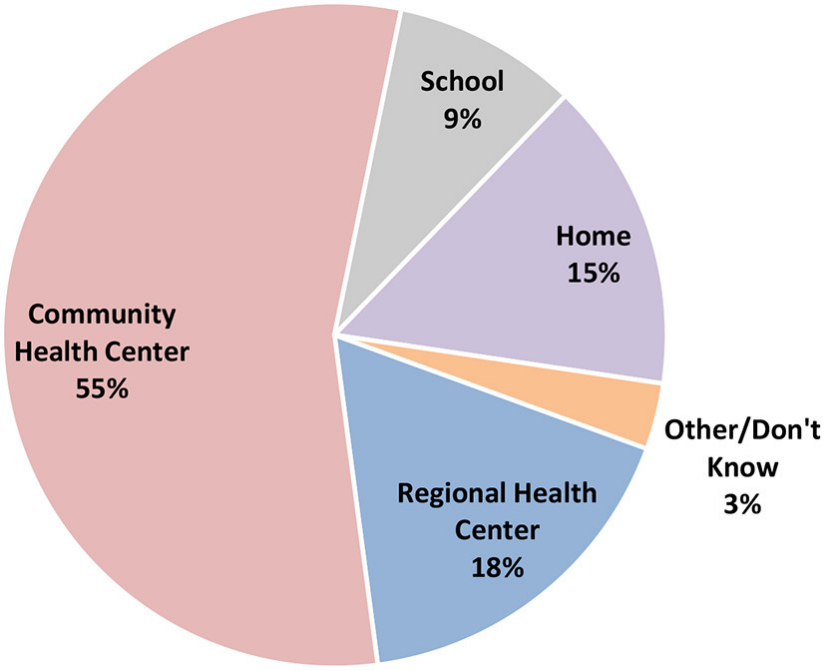
Preference regarding location of HPV vaccination. Where would you want CC vaccination to take place? This pie chart shows the distribution of preferred locations to vaccinate daughters with HPV vaccine. Most of the participants preferred to vaccinate in the local community clinics.

Finally, to assist the local health authorities with plans for future HPV vaccination campaigns, we investigated preferred locations for vaccination, preferred contact methods for 2nd dose reminders, and the best methods for obtaining parental consent. CHCs were the preferred location to vaccinate daughters as it was chosen by 55% (*N* = 344) of participants, followed by the RHC (18%, *N* = 108), home (door-to-door vaccination, 15%, *N* = 94) and school (9%, *N* = 56) (Figure 6). 73% of mothers preferred clinic-based interventions over school- or home-based interventions.

Participants also preferred to receive reminders by phone call, rather than text. While mobile phones are prevalent, many people in one family unit will often share one mobile phone, and low literacy rates may also explain the lack of enthusiasm for text messaging. When asked which member of the household would give permission and make the decision to vaccinate children, 33% (*N* = 163) participants responded themselves, 35% (*N* = 177) responded their husband and 32% (*N* = 159) stated that they would make the decision as a family. When participants were offered HPV vaccination for their child at a CHC during the time of the survey, 48% (*N* = 241) stated that they would make the decision themselves and 51% (*N* = 257) said that they would call their husband to make the decision.

## Discussion

If there is a single take away from this study, it is that access to health information is the key to increasing CC awareness and information delivered by peers is even more effective. This is not a new finding, but it is confirmation of other reports in the literature on community outreach for public health (23–29). Outreach generated high rates of acceptance and interest from community members. A systematic review recently found that reasons for non-participation in outreach activities may be due to stigma, confidentiality concerns, and mistrust (30). All of these were potentially mitigated by using trusted community liaisons.

Several factors contributed to the five-fold increase in CC screening we saw during this campaign. Certainly, the provision of screening supplies, healthcare professional training, and of free screening lowered the barriers to accessing CC screening, however, more than half of the 500 women surveyed specifically referenced an education session (either at the clinic or in their neighborhood) as the reason for seeking CC screening that day. We believe that the story-telling cloth approach used in this campaign was particularly effective because it allowed a predominately illiterate group to access information both visually through the cloth and verbally through the women wearing the cloth. Further, the results of our survey also show that the use of the story-telling cloth as a visual aid during formal and informal education sessions in the community proved more effective than prevailing outreach methods such as TV and radio ads. As the information about CC screening was transmitted in group meeting settings, by trusted local healthcare workers (and peers) who were wearing the “message” on their traditional outfits, and was combined with specific information about how to obtain CC screening in their local clinics, it is likely that social cognitive theory could explain the positive outcomes that were observed (31). This style of community-led education through story-telling cloth may serve as a model for other disease awareness campaigns in this region. GAIA VF has followed up this campaign with a similar project, to educate 14 different communities in Mali about COVID-19 risks and vaccines, in 2021. While the story-telling cloth approach may not be appropriate for more literate populations, the method appears to be adaptable for use in West African health campaigns, where literacy is low, and messages are often provided using illustrations rather than text.

It is important to note the dramatic difference between participants knowledge of HPV and of CC after the campaign. While 75% of women said that they had heard of CC {a huge improvement over the 55% [*N* = 41/75] reporting this knowledge in GAIA VF's previous KAP studies (15, 16)}, only 13% knew what HPV was (Table 2). Further, only 45% of women agreed that HPV causes CC. This finding is particularly important given that the vaccine against CC is often referred to as the HPV vaccine since it protects against the strains of HPV that causes CC. Women wanted the vaccine that protects against CC, but many women were unfamiliar with HPV and therefore resistant toward an HPV vaccine until the link between HPV and CC was explained. This suggests that it is important to leverage CC awareness as well as explain the link between HPV and CC, to improve uptake of HPV vaccines in future campaigns (32, 33).

While it is critical to increase knowledge about HPV and CC as a first step toward improving attitudes and behaviors surrounding vaccination and screening, it is also important that future programs work to expand access to those services for women who are interested. This study and various others have shown that having higher levels of education in general and knowledge about HPV/CC increase willingness to receive vaccination. However, it has also been shown that vaccination rates in low-resource settings remain low despite willingness to vaccinate due to high cost and limited access to healthcare providers (34–36). In Pakistan, many young adult respondents believed the vaccine to be costly and time-consuming (37). A systematic review of the HPV immunization literature from sub-Saharan Africa concluded that systems-level constraints on service delivery and healthcare worker capacity remain barriers to effective vaccination (38). While education is an important first step and the data in this study show that change in behaviors could be achieved, other studies have shown that education alone is not always sufficient (39).

Shortly after completion of this program, a national pilot campaign for HPV vaccination (funded by GAVI) started in Mali, targeting girls registered in school. A recent study in Brazil showed that school was the major source of HPV knowledge for adolescents as opposed to health care providers for adults, and that knowledge in this age group was less likely to be adequate compared to adults (40). This suggests that schools may represent an important opportunity throughout the world to disseminate health information to children and teens. However, in a country like Mali where an estimated 51% of girls are out of school due to poverty or the need for their help at home (41), this vaccination strategy might not as effective as it seems to be elsewhere (42). The results of our survey suggest that parents are aware of this as 73% of mothers preferred clinic-based interventions over school- or home-based interventions.

Given the relatively high proportion of women who were not comfortable making the decision to vaccinate without their husbands, methods to engage the whole family should be explored for future campaigns. This finding is in line with prior studies which have shown that support from husbands is an important predictive factor in the vaccination of married women (43). Furthermore, prior studies have shown that caregiver opinions do impact adolescent vaccination uptake but that the knowledge and biases of adolescents themselves play an important role as well (44). This suggests that in addition to disseminating information to adult women, important strides could be made if men and children were also included in educational campaigns. In addition, vaccinating male adolescents is an important part of keeping women safe from cervical cancer that has not yet been included in HPC vaccination campaigns in West Africa. Interestingly, a study from Kenya showed that most survey respondents were opposed to vaccinating boys (45).

The intervention team encountered three important challenges during this campaign. First, during the program there was a high number of women who were lost to follow-up after a positive CC screen. This may have been due to individual factors such as lack of transportation, fear of outcome of a second screen, or lack of understanding. When a woman received a positive CC screening at the community clinic, she was directed to another center for a confirmation screening, which if positive would result in a biopsy at that same appointment. This biopsy was delivered to the National Laboratory for analysis and the results were sent to the doctor after 2 weeks. Finally, upon receipt of the results, the doctor would call the patient to notify her and schedule free follow-up care, including cryotherapy or a loop electrosurgical excision procedure to remove precancerous lesions. Despite access to free follow-up and free treatment, patients were “lost to follow-up” at each of these stages.

Navigating various institutions within the city of Bamako was a potential barrier for follow-up care, due to distance or cost of travel. To improve follow-up, a system of telephone reminders was implemented a few months into the campaign period. This important challenge should inform future healthcare campaigns in low-resource settings; the programs should be designed to both remind patients to follow-up with their doctor and provide transportation support for patients being asked to seek follow-up care at a health center further from home.

Second, the timing of this campaign overlapped with the holy month of Ramadan. Mali's population is 95% Muslim, and many individuals refrain from all non-essential medical procedures during this month of fasting (46). As a result, GAIA VF's team observed a marked reduction in screening levels during this month.

Our final notable challenge was that middle-aged women (~35+ years) were underrepresented among women screened during the campaign. According to the World Health Organization (WHO), the target age group for CC screening is 30–49 years (47), but Malian national protocol encourages cervical cancer screening starting at a young age (anyone sexually active is eligible). For this reason, the campaign was not limited by age, and most of the women screened were under 30 with only 9% participants over 50 (*N* = 295/3,271), 48% under 30 (*N* = 1,557/3,271), and 43% in the WHO target age range of 30–49 (*N* = 1,419/3,271).

Physician referrals (17%) successfully brought in an older cohort of women who are statistically more at-risk for CC and had not been exposed to the other outreach methods. The mean age among “physician-referred” women was 35.2 while the mean age among those who were not referred was 30.5.

Anecdotally, community health workers during this campaign commented that older women misunderstood their level of risk for cervical cancer development believing that they were no longer at risk if they were not sexually active. GAIA VF attempted to target older women by employing middle-aged, female community health workers, but even more effort is needed to explain the specific risk of cervical cancer to this demographic. A possible way to increase participation could be to emphasize during CC education sessions that CC onset can happen even 10–20 years after exposure to HPV, and that prior sexual activity is a risk factor.

Limitations to the study include the focus on low-literacy populations (the approach would have to be modified for high literacy populations). Although a link was demonstrated between the observed effect and the Story-telling Cloth, other educational sessions might have also contributed to motivating women to get screened. Similarly, the cost reduction (two dollars per screen) test was waived during the intervention, and this may have been a motivating factor. Despite these limitations, the use of culturally appropriate and affordable local materials as a focal point for education sessions, when combined with a community-led health education campaign to inform the communities about CC prevention methods appeared to contribute to a five-fold increase in CC screening participation. The simplicity of the approach and the relatively low cost of the intervention, improves the potential for the program to be scaled up in several West African countries in conjunction with the introduction of the HPV vaccine.

## Conclusion

All in all, with a budget of only $150,200, this campaign was able to engage and educate healthcare workers to motivate 3,271 women to visit a participating clinic for CC screening, while a mere 600 women had been screened during the previous year. For comparison, a Bamako-wide campaign that improved the accessibility of cervical cancer screening using a push-pull method (SMS texting, Radio and Television, and weekend CC screening clinics), in 2016 and 2020 -was able to increase the rate of CC screening by a similar amount (in a much larger population) (48).

In summary, this study shows that a comprehensive, visual, community-centered education campaign coupled with coordinated support for local clinics improves prevention of CC. Our survey also provides some important insights about the responsibility of the parent for making decisions, the desired location of vaccination and potential motivations for vaccination, that will be useful for future vaccination programs.

## Data availability statement

The raw data supporting the conclusions of this article will be made available by the authors, without undue reservation.

## Ethics statement

The studies involving human participants were reviewed and approved by the Ethical & Independent Review Services in the United States, and the Ethical Committee of the University of Sciences, Techniques, and Technologies, Bamako, Mali. Written informed consent to participate in this study was provided by the participant herself or the participants' legal guardian/next of kin. Written informed consent was obtained from the individual(s) for the publication of any potentially identifiable images or data included in this article.

## Author contributions

All authors: study design, implementation, data collection, analysis, and/or manuscript writing and editing. All authors contributed to the article and approved the submitted version.

## Footnotes

^1^See Supplementary Tables S1, S2 in for factor analysis output and regression models run with factor instead of additive knowledge variables. Factor analysis is a statistical method for combining multiple variables into a single “factor” variable that encompasses the concept you are trying to measure with those variables. See https://stats.oarc.ucla.edu/spss/seminars/introduction-to-factor-analysis/a-practical-introduction-to-factor-analysis/ for a brief overview.
