# Supplementary material for: A story-telling cloth approach to motivating cervical cancer screening in Mali
Source: Front Public Health. 2022 Dec 15;10:1051536. doi: 10.3389/fpubh.2022.1051536 (PMC9800037; doi:10.3389/fpubh.2022.1051536)
Supplement: Supplementary file 1 [file Data_Sheet_1.docx]

Supplemental Material for Crippin et al. Story-Telling Cloth to Prevent Cervical Cancer

**S1: Story-telling Cloth Survey (English version).**

The survey was translated into French but read in Bambara to participants who could not read.

The survey is seven pages long.

**S1: Story-telling Cloth Survey (English version), page 2.**

**S1: Story-telling Cloth Survey (English version), page 3.**

**S1: Story-telling Cloth Survey (English version), page 4.**

**S1: Story-telling Cloth Survey (English version), page 5.**

**S1: Story-telling Cloth Survey (English version), page 6.**

**S1: Story-telling Cloth Survey (English version), page 7.**

**S2: Description of the training that took place prior to intervention (in French)**

RAPPORT DE LA FORMATION DES PRESTATAIRES DU DISTRICT DE BAMAKO SUR LE DEPISTAGE DU CANCER DU COL DE L’UTERUS

L’an 2015 du 30 Mars au 04 Avril s’est déroulé dans la salle de réunion du CSREF CI l’atelier de formation du personnel du CSRéf Commune I et des CSCom (ASACODJe, ASACOBoul1, ASACOBA, ASAComsi) de la commune 1 sur le dépistage du cancer du col par des tests visuels (IVA, IVL) dans le cadre du projet GAIA.

Etaient présents :

- Au niveau CSCOM : un médecin, une sage-femme, une infirmière obstétricienne, une matrone ;
- Au niveau CSREF : 03 sage femmes et une infirmière obstétricienne.

La facilitation était assurée par 2 facilitateurs de la DRS et un facilitateur du CSREF de la commune I.

Les cérémonies d’ouverture et de clôture étaient présidées par le médecin chef de la commune 1.

Le but de l’atelier était de renforcer les compétences des prestataires en dépistage du cancer du col d l’utérus.

Les objectifs étaient entre autres :

-Identifier les différentes méthodes de diagnostic du cancer du col de l’utérus disponibles au Mali.

-Préparer les réactifs pour le dépistage par les teste visuels.

-Reconnaitre les anomalies des tests d’inspections visuels

-Expliquer le traitement des lésions précancéreuses du col de l’utérus.

LA METHODOLOGIE UTILISEE ETAIT :

- Exposées illustrées ;

-Projection par diapo des lésions colposcopiques.

-Travaux pratiques.

-Brain storming.

Au cours de cet atelier différents thèmes ont été étudié :

-L’anatomie de l’appareil génital de la femme.

-Des lésions précancéreuses du col utérin.

-Le dépistage du cancer du col utérin.

-La préparation des réactifs (acide acétique 4% et le Lugol) et leur conservation.

-Les matériels pour le dépistage.

-L’introduction à l’IVA et IVL.

-Présentation par diapo des lésions colposcopiques.

-Vidéo sur la colposcopie.

-Exercice sur le dépistage.

-Présentation des supports du projet GAIA.

Les travaux pratiques sur le terrain ont permis à chaque participant de réaliser au moins 05 dépistages pendant les 04 jours selon les objectifs du projet.

Les discussions ont porté sur :

- Les différents types de coloration a l’acide acétique (blanc opaque en contact avec la nouvelle ligne de jonction et située dans la zone de remaniement) et au Lugol (coloration jaune moutarde en contact avec la nouvelle ligne de jonction et située dans la zone de remaniement)
- L’ancienne et la nouvelle ligne de jonction
- La zone des transformations (métaplasie, dysplasie)
- la conservation des réactifs.

Les résultats du stage pratique :

| BOUL I | |
| --- | --- |
| Positifs | 10 |
| Négatifs | 53 |
| Ectropion | 3 |
| Cervicite | 4 |
| Total | 70 |
| CSRéf | |
| Positif | 0 |
| Négatifs | 34 |
| Cervicite | 2 |
| Ectropion | 0 |
| Total | 36 |
| COMSI | |
| Positif | 2 |
| Négatifs | 25 |
| Cervicite | 1 |
| Ectropion | 0 |
| Total | 27 |
| TROIS SITES | |
| Positif | 12 |
| Négatifs | 112 |
| Cervicite | 7 |
| Ectropion | 3 |
| TOTAL GENERAL | 134 |

Au cours de cet atelier les participants ont formulé quelques recommandations :

CSCom :

-Intégrer l’activité de dépistage dans le PMA ;

-formation continue et restitution aux autres personnels du centre.

DRS/CSRef :

-Assurer la supervision formative des CSComs ;

-Formation recyclage du personnel des CSComs.

GAIA :

-Assurer la dotation continue des réactifs et consommables.

-Fournir les supports

-Améliorer la motivation du personnel impliqué

Bamako le 04/04/2015

RAPPORT DE FORMATION DES PAIRS EDUCATEURS (RELAIS) SUR LA PREVENTION, L’EDUCATION A LA SANTE ET CONSEIL RELATIF A LA LUTTE CONTRE LE CANCER DU COL DE L’UTERUS DANS LA COMMUNE 1 DE BAMAKO.

L’an 2015 du 07 au 09 Avril s’est déroulé dans la salle de réunion du CSREF CI la formation des pairs éducateurs (relais) sur la prévention, l’éducation à la sante et conseil relatif à la lutte contre le cancer du col de l’utérus dans la commune 1 de Bamako de 4 aires de sante (ASACODJe, ASACOBoul1, ASACOBA, ASAComsi).

Etaient présents:

- Au niveau des aires de sante : 4 relais par aire de sante ;
- La facilitation était assurée par 2 facilitateurs de la DRS et un facilitateur du CSREF de la commune I.

Les cérémonies d’ouverture et de clôture étaient présidées par le médecin chef de la commune 1.

Le but de l’atelier était de renforcer les compétences des prestataires prévention, l’éducation à la sante et conseil relatif à la lutte contre le cancer du col

***2. Objectifs de la formation***

**Les objectifs éducationnels**

1. Être en mesure de dispenser au sein de la communauté et des structures sanitaires une information correcte relative au cancer du col de l’utérus à l’aide du pagne qui parle.
2. Être en mesure d’organiser des séances d’animation pour sensibiliser la communauté sur le cancer de l’utérus en vue de réduire la morbidité et les décès associés à cette maladie.
3. Fournir à la population cible des renseignements cohérents et corrects sur l’intérêt du dépistage du cancer du col de l’utérus et d’une prise en charge précoce des cas.

***3. Méthode d’enseignement :***

La méthodologie participative sera privilégiée pour animer la formation. D’autres approches comme des questions/réponses, exposés et des travaux de groupe seront aussi utilisés.

Au cours de cet atelier différents thèmes ont été étudies :

-L’anatomie de l’appareil génital de la femme ;

-Des lésions précancéreuses du col utérin ;

- Les messages clés dans le cadre de la prévention de lutte contre le cancer du col de l’utérus ;

- Les techniques d’animation et de communication en matière de prévention du cancer du col de l’utérus ;

- Les outils de communication adaptés au contexte local dans le cadre de la prévention du cancer du col de l’utérus.

- Présentation des supports du projet GAIA.

Les discussions ont porté sur :

- Les différents types Les messages clés dans le cadre de la prévention de lutte contre le cancer du col de l’utérus ;
- Les messages du pagne du projet GAIA ;
- Les intérêts de se faire dépister pour les femmes.

Au cours de cet atelier les participants ont formulé quelques recommandations :

CSCom :

-Intégrer les résultats des activités de dépistage de relais dans le rapport du CSCom.

DRS/CSRef :

-Assurer la supervision formative des relais.

GAIA :

-Fournir les supports ;

-Améliorer la motivation du personnel impliqué.

Bamako le 09/04/2015

# S3: Supplemental Tables

**S.1 Factor Analysis Output**

| Number of Observations = 500  Retained factors = 2  Chi^2^=442.17, p<.001 |
| --- |
| Factor \| Eigenvalue Difference Proportion Cumulative |
| Factor 1 \| 2.34782 1.25902 0.3354 0.3354 |
| Factor 2 \| 1.08880 0.15620 0.1555 0.4909 |
| Factor 3 \| 0.93260 0.07982 0.1332 0.6242 |
| Factor 4 \| 0.85278 0.14969 0.1218 0.7460 |
| Factor 5 \| 0.70309 0.12611 0.1004 0.8464 |
| Factor 6 \| 0.57698 0.07906 0.0824 0.9289 |
| Factor 7 \| 0.49792 . 0.0711 1.0000 |
| Notes: Factor 1 was retained for comparison to additive *knowledge* DV presented in paper. See results of identical regression using Factor1 instead of the additive *knowledge* in linear regression knowledge below. Substantive results remain the same. |

**S.3 Regression Models using Factor Analysis for comparison to Additive *Knowledge* Variable.**

|  | Model 1 | Model 2 |
| --- | --- | --- |
|  | *DV=Factor1 for Knowledge* | *DV=Factor1 for Knowledge* |
|  |  |  |
| Attended an Education Session | 0.26* | 0.302** |
|  | (0.098) | (0.087) |
| Saw Story-telling Cloth | 0.314** | 0.261* |
|  | (0.137) | (0.108) |
| Constant | -.676** | -.814** |
|  | (0.192) | (0.11) |
|  |  |  |
| Observations | 487 | 487 |
| R-squared^+^ | 0.264 | 0.289 |
| Method = Linear Regression. DV coded -1.12 to 3.03 from less to more knowledge.  Control Variables not displayed since this table is just for comparison.  Robust standard errors clustered by site in parentheses  *** p<0.01, ** p<0.05, * p<0.1  **Interpretation:** The positive coefficients mean that the independent variable has a positive effect on *knowledge* and negative coefficients mean they have a negative effect. A 1 unit change in *Attended an Education Session* results in a .26-point increase in the factor | | |

| **S.4 Ordered Logit Models for Comparison to Linear Regression Results** |
| --- |
|  |

|  | Model 1 | Model 2 |
| --- | --- | --- |
|  | *Ordered Logit* | *Ordered Logit* |
|  |  |  |
| Attended an Education Session | 0.918*** | 0.930*** |
|  | (0.330) | (0.333) |
| Saw Story-telling Cloth | 0.863*** | 0.799*** |
|  | (0.285) | (0.300) |
| Age | 0.008 | 0.014 |
|  | (0.012) | (0.011) |
| Married – Monogamous ^a^ | -0.364 | -0.379 |
|  | (0.395) | (0.386) |
| Married – Polygamous ^a^ | -0.266 | -0.272 |
|  | (0.550) | (0.525) |
| Schooling | 0.261*** | 0.273*** |
|  | (0.0983) | (0.102) |
| Ever Been Pregnant | -0.0438 | -0.116 |
|  | (0.339) | (0.291) |
| Ever had STI | 0.699** | 0.698** |
|  | (0.333) | (0.346) |
| Ever been tested for HIV | 0.752*** | 0.723*** |
|  | (0.260) | (0.270) |
| Ever had a Pelvic Examine | -0.321 | -0.356 |
|  | (0.355) | (0.420) |
| Ever been screened for CC | 1.152*** | 1.111*** |
|  | (0.279) | (0.314) |
| Know someone who had/has CC | 0.944*** | 0.958*** |
|  | (0.195) | (0.196) |
| ASACOBOUL1 ^b^ |  | 0.177 |
|  |  | (0.222) |
| ASACODJE ^b^ |  | 0.456*** |
|  |  | (0.108) |
| ASACOMSI ^b^ |  | 0.028 |
|  |  | (0.192) |
| CSREF ^b^ |  | -0.107 |
|  |  | (0.159) |
| Constant |  |  |
| Observations | 487 | 487 |
| Log- Pseudo Likelihood | -857.27 | .0969 |
| ^a^ Excluded Group = Unmarried. ^b^ Excluded Group = ASACOBA.  Robust standard errors clustered by site in parentheses  *** p<0.01, ** p<0.05, * p<0.1 | | |
